# Automatic dispersion, defect, curing, and thermal characteristics determination of polymer composites using micro-scale infrared thermography and machine learning algorithm

**DOI:** 10.1038/s41598-023-29270-z

**Published:** 2023-02-16

**Authors:** Md Ashiqur Rahman, Mirza Masfiqur Rahman, Ali Ashraf

**Affiliations:** 1 Department of Mechanical Engineering, University of Texas Rio Grande Valley, Edinburg, TX 78539 USA; 2 Department of Computer Science, Purdue University, West Lafayette, IN 47907 USA

**Keywords:** Two-dimensional materials, Characterization and analytical techniques, Imaging techniques, Computer science

## Abstract

Infrared thermography is a non-destructive technique that can be exploited in many fields including polymer composite investigation. Based on emissivity and thermal diffusivity variation; components, defects, and curing state of the composite can be identified. However, manual processing of thermal images that may contain significant artifacts, is prone to erroneous component and property determination. In this study, thermal images of different graphite/graphene-based polymer composites fabricated by hand, planetary, and batch mixing techniques were analyzed through an automatic machine learning model. Filler size, shape, and location can be identified in polymer composites and thus, the dispersion of different samples was quantified with a resolution of ~ 20 µm despite having artifacts in the thermal image. Thermal diffusivity comparison of three mixing techniques was performed for 40% graphite in the elastomer. Batch mixing demonstrated superior dispersion than planetary and hand mixing as the dispersion index (DI) for batch mixing was 0.07 while planetary and hand mixing showed 0.0865 and 0.163 respectively. Curing was investigated for a polymer with different fillers (PDMS took 500 s while PDMS-Graphene and PDMS Graphite Powder took 800 s to cure), and a thermal characteristic curve was generated to compare the composite quality. Therefore, the above-mentioned methods with machine learning algorithms can be a great tool to analyze composite both quantitatively and qualitatively.

## Introduction

Polymer composites are comprised of two or more materials (matrix and filler/reinforcing/additive materials) that have properties that are superior to the properties of the individual materials^1–3^. Because of its synergistic properties and applications in aerospace, automotive, maritime, energy, and consumer fields, it has attracted the interest of both industry and academia^4–8^. Among all the fillers or reinforcing materials, graphite or graphene has become an ideal candidate due to its exceptional mechanical, thermal, and electrical properties. Thus, graphene-based polymer composites have captured the scientific community's interest during the past few decades.

The properties of polymer composites largely depend on the dispersion of filler materials on the polymer matrix. Thus, the performance of a polymer composite (poor or good) is determined directly by the degree of agglomeration, which can lead to property variation over the composite. The study of particle/loading size, shape, and size can be accomplished using transmission electron microscopy (TEM)^9^, but it is restricted to relatively smaller samples. Scanning electron microscopy can be another technique to determine dispersion, and Fu et al*.* calculated the carbon nanotube (CNT) dispersion index by dividing the images into grids^10^. The majority of TEM and SEM procedures, which are expensive and require a complex sample preparation process (sample preparation might be destructive), are employed to estimate the dispersion of low-weight percentage of filler materials on a smaller scale qualitatively^11^.

Another challenge to use polymer composite extensively is to develop a non-destructive method to check the quality/performance of the composites. The ultrasonic method (impulse acoustic microscopy) was used to investigate the filler distribution or microstructure in carbon nanocomposite specimens prepared using a traditional method and a vacuum mixer^12^. The potential applications in industry are, however, constrained by this non-destructive evaluation (NDE) technique's slowness in sample preparation and ability to scan only smaller samples^13^.

Developing an NDE process for measuring the dispersion quantitatively rather than qualitatively, particle/filler size, shape, and agglomeration can be an excellent technique for predicting polymer composite performance. Infrared (IR) thermography is a non-contact method of measuring temperature variation that analyzes the infrared radiation emitted by an object^14^. Among different thermography methods, active thermography (external excitation of the sample) is generally used to detect the surface/sub-surface defect in fiber-reinforced composites or concrete structures^15^. The surface temperature obtained via active IR thermography (a few mm depth) can lead to the determination of the internal temperature of composites (heat transfer modeling along the entire depth), thus composite quality can be determined^16^. For in-depth analysis, lock-in thermography can be a useful approach, however, the operator needs to change the thermal excitation frequency for testing^17,18^. In recent years, composites with nano/micro-sized fillers have emerged significantly, emphasizing the need for microscale thermography. Therefore, infrared active thermography performed at micro-scale can be a useful technique for measuring the dispersion of nano/micro fillers. For instance, infrared thermography was used by Pantano et al*.* to evaluate the poor dispersion of carbon nanotubes in nanocomposites^19^. Ashraf et al*.* studied the dispersion (quantified as dispersion index) and thermal properties of graphene polymer composites using a close-up lens infrared thermography^20^. Gresil et al*.* studied the thermal diffusivity mapping of graphene-based polymer nanocomposites at a resolution of 200 µm per pixel^21^. Void or flaw detection is also determined for graphene-based composite via infrared thermography^22^. However, the process mentioned above to determine the filler/void/flaw shape and size is manual and thereby takes a lot of time in the manufacturing line. Additionally, defocused images or images with artifacts/void/tramp material sometimes provide wrong information about the sample quality. So, automatic detection of fillers, voids, flaws, and artifacts should be employed for accurate results. To the best of our knowledge, automatic dispersion/flaw/void/tramp material quantification of composites has not been reported yet by the scientific community.

After producing thermal images from each experiment one can quantify dispersion by color thresholding each image using image processing. However, to leverage such downstream quantification, unrealizable human effort is required; needless to mention the significant risk of producing erroneous results. Often, it is impossible to correctly quantify dispersion from noisy or incomplete data. Artifacts essentially pose a great threat in quantification since it is difficult to differentiate them from nano-fillers in a thermal image frame unless one has complete access to subsequent (or previous) image frames of the experiment. The recent development of image processing techniques, mostly available through get-go libraries in MATLAB, Python, or R; has been quite useful in such events, although it neither reduces the manual involvement nor ensures accuracy. A more recent effort to incorporate machine learning towards automated image processing tasks such as image segmentation, noise removal, object detection, recovery, etc. has demonstrated success^23–30^ and it begs an evaluation for dispersion quantification in a similar manner. We present a Surface Dispersion quantification method using Fourier Neural operator, SDFN, that does few-shot learning on thermal data and automatically quantifies dispersion when presented with data from unseen experiments.

The inclusion of numerous additives (such as promoting agents, fillers, etc.) in commercial formulations leads to complex cure kinetics, making a thorough understanding of curing the most important prerequisite for the optimization of composite processes^31^. By heating the sample from the ambient condition, the temperature rises to a certain maximum point before curing (depending on filler materials), followed by a sudden temperature drop. The reaction rate or curing kinetics changes due to filler influence (change in the rate constants)^32^. Thus, recording the temperature change with respect to time can provide information about the curing (micro-scale/bulk curing analysis), which is difficult to achieve by conventional methods such as differential scanning calorimetry (DSC).

In this study, we performed dispersion analysis via micro-scale IR thermography in a wide range of samples prepared by hand, planetary, and batch mixing. Different mixing techniques can significantly alter dispersion efficiency and therefore composite properties. These samples were analyzed using SDFN and thus, it made it possible to quantify the filler amount or dispersion of polymer composites efficiently. Thermal diffusivity quantifies the speed of heat transfer in a sample, and the diffusivity of polymer composites is influenced by dispersion/homogeneity. So, thermal diffusivity mapping and therefore dispersion index of different mixing techniques were compared. Finally, curing analysis of polymer with different fillers and thermal characteristics of hand, planetary, and batch mixing samples were investigated.

## Materials and methods

### Materials

Dragon skin and Ecoflex 00-30 (platinum-catalyzed silicones) were purchased from Smooth-On (USA), and Graphite flake (average size: + 20 mesh (850 microns)) was obtained from Asbury Carbons (USA). Molybdenum Disulphide (MoS_2_-Powder size 1.5 μm) was provided by ACS Materials LLC. SYLGARD™ 184 Silicone Elastomer Kit was obtained from Dow Corning. Graphene nanoplatelets (surface area 750 m^2^/g, size ~ 2 μm) and graphite powder (~ 20 μm) were acquired from Sigma Aldrich (USA) and Fisher Chemicals respectively.

### Methods

Dragon skin both part A and part B were mixed with graphite (G) flake with a 2.5, 5, 7.5, and 10 weight percent. Graphite with part A and part B was mixed in a 1:1 ratio through simple hand mixing and high-speed planetary shear mixing techniques (two different procedures result in different dispersion in polymer composites). Another mixing of a higher percentage of graphite (40% G) in Ecoflex part A and part B both was done in a Randcastle batch mixer, where bulk graphite is exfoliated to graphene via shear exfoliation^33^ (two samples were prepared, first sample is 100 rpm and 3 min of mixing, second one is 100 rpm and 10 min of mixing). Simple hand mixing, planetary mixing (mixed at 2000 rpm for 1 min) and batch mixing samples will be referred to as 2.5/5/7.5/10% G Hand, 2.5/5/7.5/10% G Planetary, and 40% G Batch throughout this manuscript respectively. The specification for the infrared thermal camera using in this project are: Camera—Fluke RSE600 Mounted Infrared Camera, Resolution—640 × 480, Frame Rate: 60 Hz, Field of view—34°H × 25.5°V, Thermal sensitivity ≤ 0.040 °C at 30 °C target temp (40 mK).

**Figure 1:**
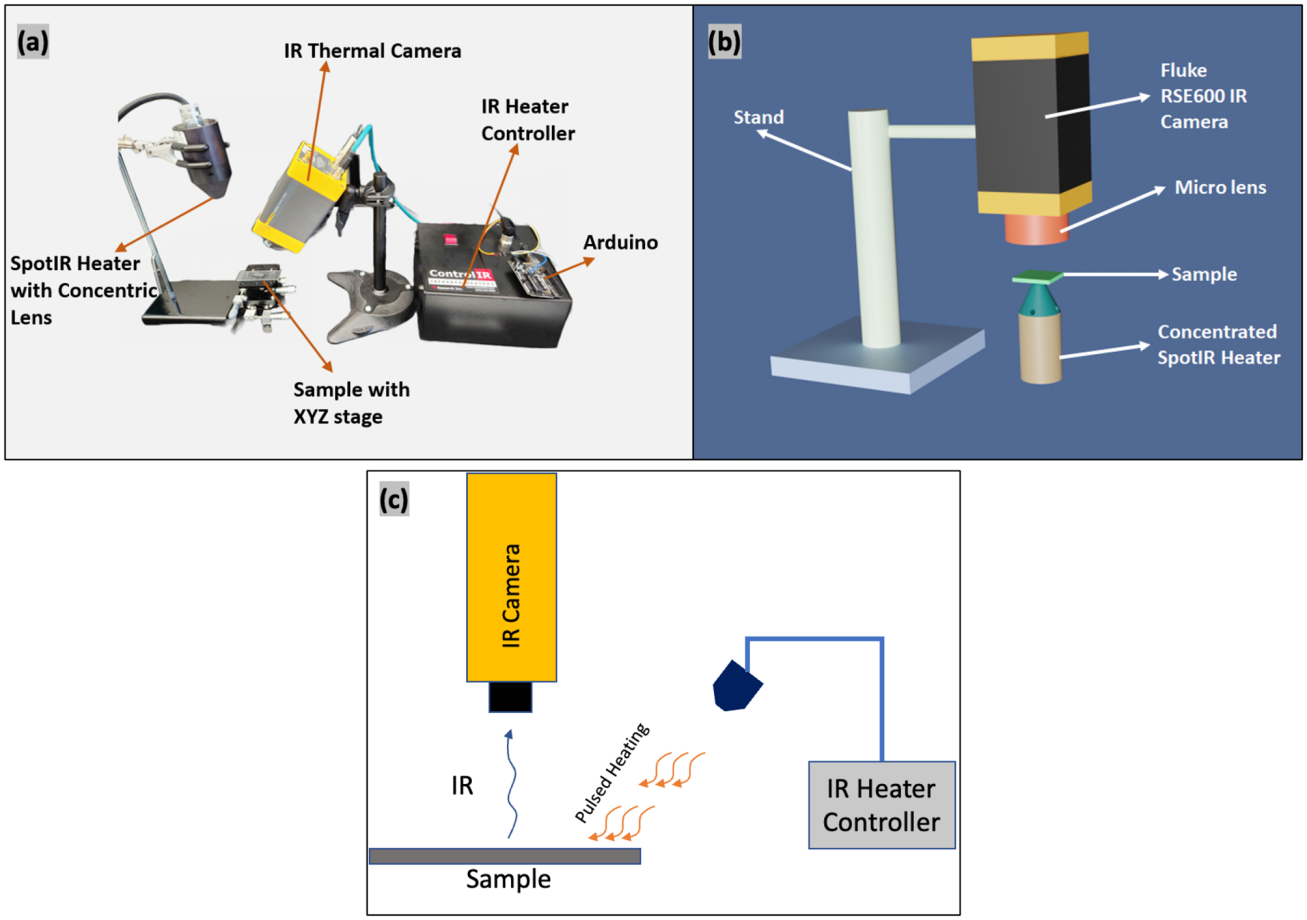
(**a**) Setup for dispersion and thermal characteristics analysis for polymer composites via infrared thermography, (**b**) schematic of thermal diffusivity testing for polymer composites, (**c**) schematic of IR heating on a sample surface and IR capturing via Fluke RSE600 thermal camera.

For dispersion and thermal characteristics analysis using IR thermography, both hand mixed and planetary-mixed 2.5–10% G samples (sample size 15 × 8 mm^2^, thickness 1.5 mm) were heated using a SpotIR heater and cooled for 30 s in ambient air. Additionally, for higher range dispersion analysis, two 40% G Batch mixing samples (sample 1 and sample 2) were analyzed. Finally, thermal diffusivity mapping of 40% G Batch samples, 40% G Planetary and 40% G Hand, 10% G (Hand mixed), and 10% G Planetary samples were carried out via modified ASTM E1461-Standard Test Method for Thermal Diffusivity by the Flash Method^34^). Using a laser source for heating the sample is costly and exposure to the laser can cause several injuries. So, instead of using a laser source, we adopted a SpotIR Model 4150 heater that comes with concentrated heating option (contains an elliptical reflector, spot diameter ~ 0.25 inch/6.4 mm). A controller Model (Model 5420 mA Power Controller) was used to control the power from 0 to 100%. An Arduino was used to provide 4–20 mA current signal to run the controller, and that provided the equivalent power (0–100%). A pulse duration of 200 ms was set via arduino programming for heating the sample with the IR heater. The setup for dispersion and thermal characteristics analysis and schematic of thermal diffusivity testing for polymer composites is shown in Fig. 1a,b. Figure 1c depicts the IR heating on the surface controlled by the Arduino pulse signal, and radiative and emissive IR capturing via Fluke RSE600 thermal camera. Zeiss field emission scanning electron microscopy (FESEM) was used to examine the morphology of cold fractured surfaces. Using a 50 × magnification ReniShaw inVia reflex system, Raman data was collected using a 633 nm laser.

### SDFN

As mentioned in the previous section, the aim is to understand the pattern of nano/micro fillers from thermal images. Since this is a time series data, and heat proportionally varies with time, each image essentially represents a different distribution of temperature. Fourier Neural Operator was first introduced to solve the family of PDEs^30^. Thus, to leverage its effectiveness, SDFN model was proposed, which understands the underlying nature of the material composition, not giving sole attention to the particular heat signature of that image. To train the model, 9 different experimental data was gathered with varying material composition and temperature. The idea behind generating such a dataset is that if the model needs to accurately understand and generalize the representation of nanopores in previously unseen data of varying temperatures, it must first learn from a diverse dataset. Once trained on data from these 9 experiments, the model can be deployed to quantify dispersion from a thermal image it has never seen before.

#### Fourier neural operator

The SDFN architecture is shown schematically in Fig. S1. The neural operator has 7 layers—the first two layers (P) are for high-dimensional feature representation (uplifting layers) and the last layer is for projection to the target dimension (Projection layer). The rest of the layers are each a combination of Fourier and linear sublayers. An image of size *M* × *N* × 3 is fed to the network. Here *M* is the width, *N* is the height, and 3 is the number of channels. The first two channels are a mesh representation of the *x* coordinates and the *y* coordinates of the thermal image. The third channel is the thermal signature itself. The network first uplifts this *M* × *N* × 3 image to size *M* × *N* × *W* , where *W* is a hyperparameter. This is passed through ${\mathcal{F}}$ which applies the discrte Fourier transform on this and keeps the 30 lower frequency modes from this high dimensional data. The main idea behind keeping only these lower frequency modes is to generalize well among images and avoid noises that are mostly incorporated as higher frequency. Next, it passes through layer *R*. This is the layer where the model optimizes and saves the multipliers for the Fourier components. Next, the output from the *R* layer is transformed using the inverse Fourier transform at ${\mathcal{F}}^{ - 1}$. There is also a linear layer *W* which contains weights to multiply to the raw input. The output of the linear sublayer *W* is added to the output of ${\mathcal{F}}^{ - 1}$. Later this combined output is passed through a GELU activation function, *σ*^35^. The activation function denotes the end of one Fourier layer. Finally, the output of three such layers is then fed to a projection layer Q that transforms the high dimensional data back to an *M* × *N* × 1 size image, which is our expected output, i.e., threshold images. Once this output is produced, anyone can trivially count the black pixels from it and report the surface dispersion.

## Results and discussion

### Automatic dispersion, defects (void, tramp material) analysis using image processing and machine learning

#### Dispersion analysis

Elastomer/polymer matrix has different emissivity compared with the fillers/additives added in composites. So, fillers can be detected easily by using an IR thermal camera. Through analyzing the image obtained by the IR camera, dispersion in the surface can be detected. Through image processing, quantification of dispersion is possible in real-time, and this can be a useful method to analyze filler size, shape, voids, or any tramp/foreign material present in composites. In a manufacturing or production line, this above-mentioned method can be applied to determine whether the batch is of standard quality or not.

**Figure 2:**
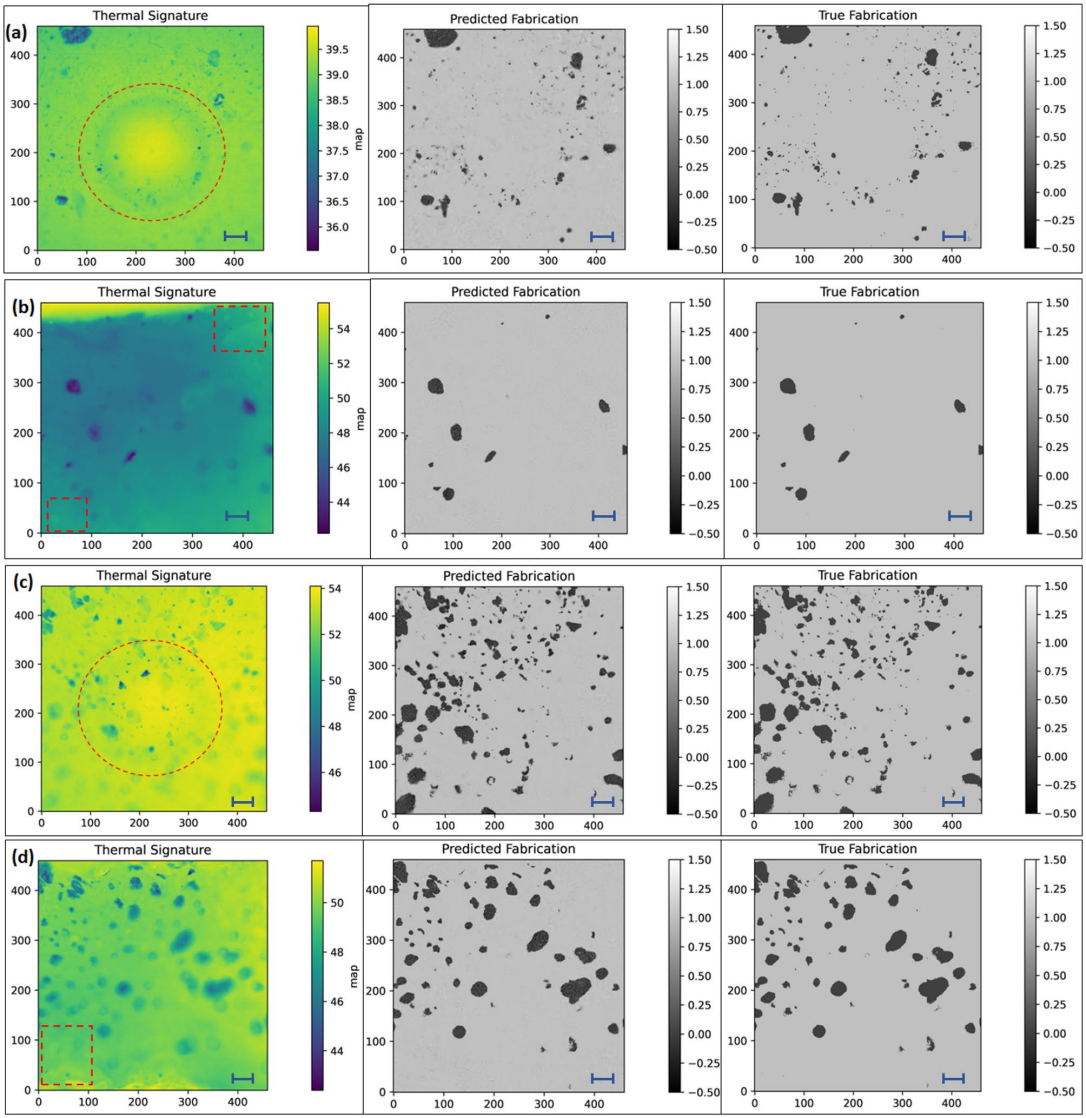
Thermal image, corresponding predicted, and true image for (**a**) 2.5% G Planetary, (**b**) 2.5% G Hand, (**c**) 10% G Planetary, (**d**) 10% G Hand. Thermal image is manually fed in image processing to remove artifacts or voids or blurriness (scale bar = 1000 microns, enclosing red circle area remarks artifact due to container mark, enclosing rectangle area remarks void area). The thermal image is then fed into machine learning model to obtain the true fabrication. By feeding a few thermal signatures, this learning method can show true fabrication in a really quick manner.

**Figure 3:**
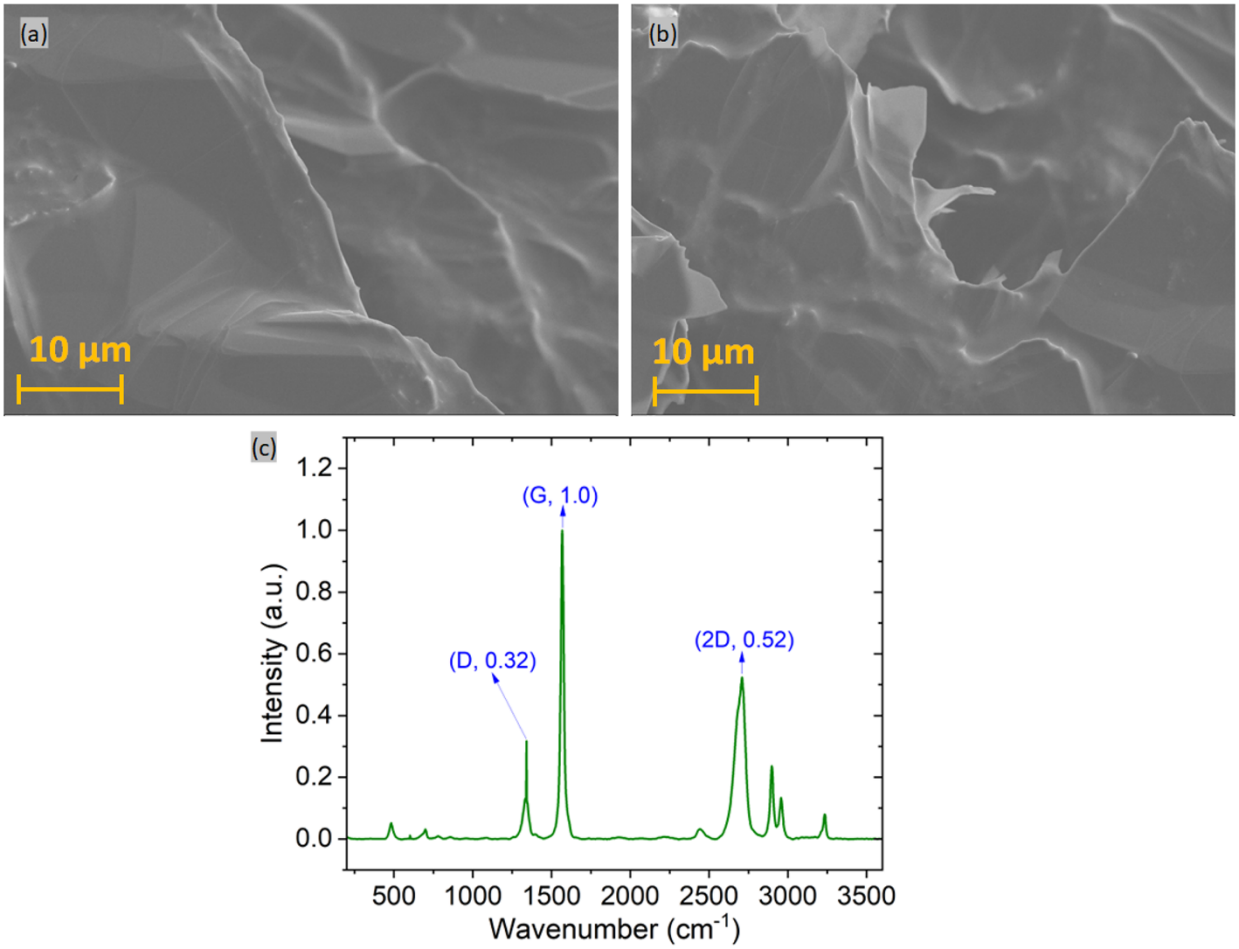
(**a** and **b**) SEM image showing graphene morphology in 40% G Batch sample at different locations, (**c**) Raman spectroscopy of 40% G Batch sample.

**Figure 4:**
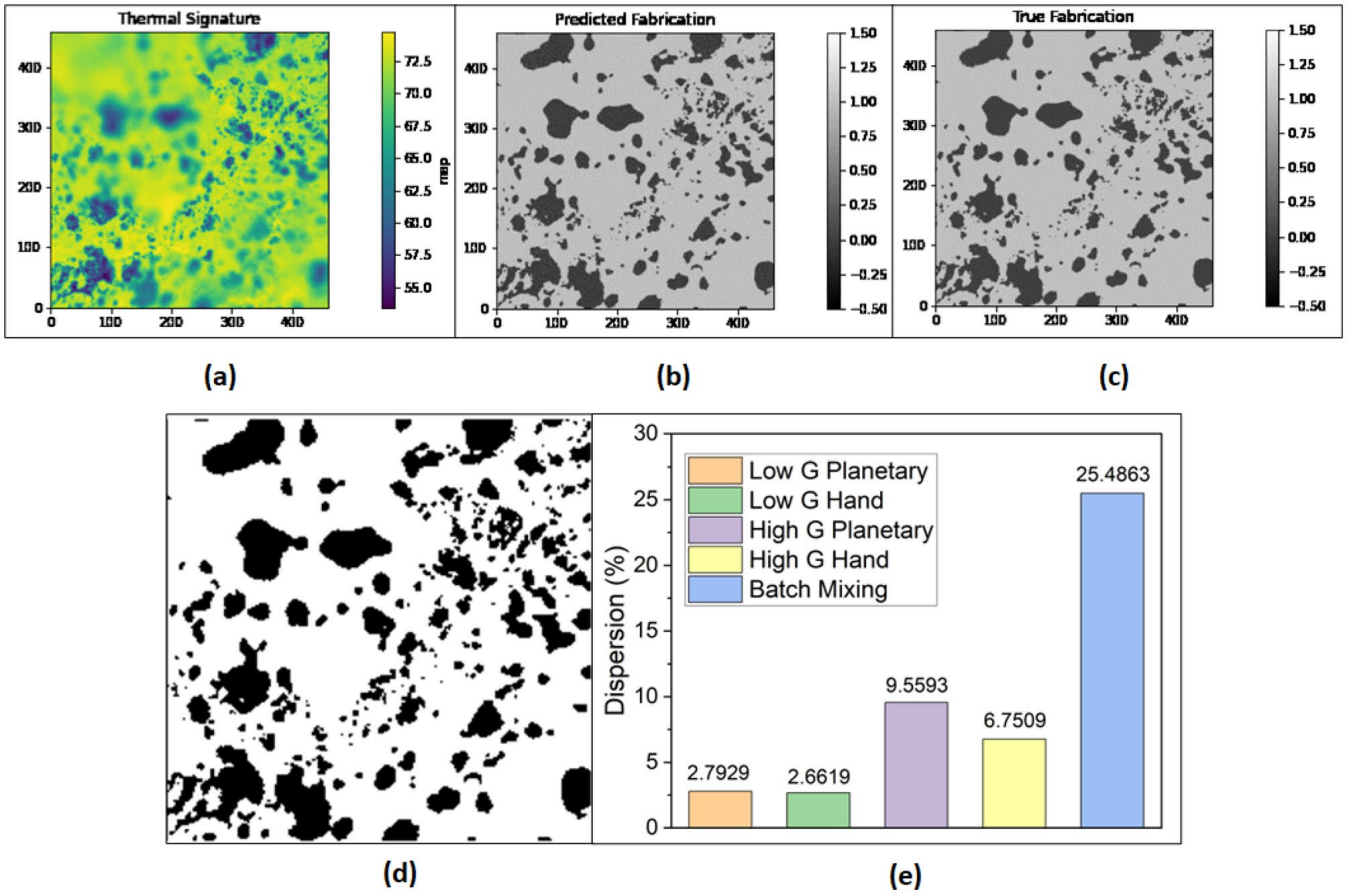
(**a**) Thermal image obtained for 40% G Batch mixing (out of focus at the top left corner), (**b**–**d**) corresponding predicted image, true and binary image obtained via machine learning and image processing (removed the blurriness due to out of focus) of the thermal image in (**a**), (**e**) Dispersion (%) of different mixing process calculated from the area pixels of the composites.

Figure 2 shows the thermal signature (temperature in each pixel) obtained from the IR camera, followed by predicted image and true image obtained from image processing and machine learning algorithm (for 2.5% G Planetary, 2.5% G Hand, 10% G Planetary and 2.5% G Hand samples). Fillers are clearly distinguishable despite of having manufacturing defects/artifacts/blurry images due to manual experimental setup and lossy generation. Then, the true image shows the dispersion which was manually produced for training the machine learning model. This is the only time the model requires this manual effort. The model was trained in 4 different setups with 50, 100, 200, and 500 data respectively. Even when trained with 50 images (on average ~ 5 images from each experiment) the predicted images reach close to the true image, although are not optimal. The test loss and training loss of the SDFN model with this varying setup of runs is shown in Fig. S2. As expected, the model trained on 500 data achieves minimum training loss (~ 0.06) and thereby converges. For optimal result, a training dataset size of 200 or 500 can be used. Additionally, 5% and 7.5% G Hand and Planetary sample thermal, predicted, and test images are shown in Fig. S3. For the higher amount of graphite and graphene content, 40% G batch mixing samples were also closely inspected. Scanning electron microscopy (SEM) images for 40% wt. G Batch was shown in Fig. 3a,b. These images indicate the graphene flakes and their shapes in different spatial locations. Raman spectroscopy provides a reliable technique to determine the number of layers and some other properties in a carbon-based material^36^. The normalized intensity of the 2D band is 0.52 in comparison to the G band (I_2D_/I_G_) (Fig. 3c), indicating that few graphene layers were formed during graphene exfoliation. So, graphite alongside graphene was also detected in thermography, and the thermal signature, and predicted images of 40% G Batch sample were shown in Fig. 4a,b. Figure 4c,d depicts the true and binary images of the sample. As the graphite/graphene was dispersed randomly, surface dispersion can be calculated by using the following formula in Eq. (1)^37^:

1 $${\text{Surface\,Dispersion}} = {\text{A}}_{{{\text{flake}}}} /{\text{A}}_{{{\text{total}}}}$$

where A_flake_ is the pixel area of the filler region (black region) and A_total_ is the total area. Surface dispersion calculated from Eq. (1) for different mixing processes (samples were prepared with low and high amounts of graphite (G) % via both hand and planetary mixing) is shown in Fig. 4e. The dispersion of Low G Hand and Planetary samples was 2.6619 and 2.7929 respectively which depicts better dispersion on planetary mixing. For High G samples, the planetary sample dispersion was 9.5593% while for hand mixing it was 6.7509%. This further illustrates the better mixing capability (less agglomeration) of planetary mixing. On the other hand, the sample fabricated from batch mixing showed a surface dispersion of 25.4863%. Thus, surface dispersion/distribution can be obtained using microscale thermography that can be useful to determine composite performance where surface phenomenon (surface wettability, conductivity) is important.

#### Defect (void/tramp material analysis)

For polymer composites, voids are typically the result of poor manufacturing or fabrication of the material, and thus it affects the mechanical properties and lifetime of the composites. It can also act as crack initiation and moisture penetration site^38^. Thus, to get the desired property of polymer composites, voids should be avoided. Analyzing the void using conventional microscopy or optical image is cumbersome, however, using infrared thermography voids can be identified as they will show a different temperature signature than fillers and polymer matrix. So, by analyzing each pixel, similar regions (void/tramp material) can be determined as the temperature will be within a specific range. To verify this hypothesis, elastomer composites with specific regions were prepared with MoS_2_ fillers, then mixed them with graphite fillers and another region with no fillers. Figure S4 shows that MoS_2_ and graphite filler regions have lower and higher average temperatures than the polymer region respectively. This is attributed to the distinctive emissivity of different regions of the sample. Thus, any tramp material that is present in a polymer composite sample thus can be identified via infrared thermography.

### Thermal diffusivity and dispersion index of polymer composites

Thermal diffusivity mapping was done using ASTM E1461-Standard Test Method for Thermal Diffusivity by the Flash Method^21,39^. For the same weight percent, the dispersion level of graphite fillers is different in samples prepared by different methods (hand, planetary, or batch mixing). Using a pulsed thermal source (SpotIR heater), the rear face temperature was recorded by a Fluke RSE600 IR thermal camera. The halftime (*t*_1/2_) required from the initiation of the pulse for the rear face temperature from baseline to its maximum is required to measure thermal diffusivity, α. Using the following Eq. (2), thermal diffusivity can be measured:

2 $${\alpha } = \frac{{0.13879L^{2} }}{{t_{0.5} }}$$

where L is the thickness of the sample. For thermal diffusivity experiment, the geometrical resolution for thermal diffusivity testing was ~ 0.1 mm/pixels. As the IR camera divides the whole image into 640 × 480 pixels, by analyzing every pixel the thermal diffusivity of the sample was determined. There could have been heat losses and non-uniform heating in our experiment because the sample was heated in an area with a diameter of around 6.4 mm. So, the relative thermal diffusivity was determined to compare the dispersion in polymer composites in a semi-quantitative approach with the aid of machine learning. Thereby, the dispersion index (DI) is calculated from thermal diffusivity to quantify the homogenization/dispersion of composites. Dispersion index is given by (Eq. 3):

3 $${\text{DI}} = { }1 - \frac{{{\upalpha }_{min} }}{{{\upalpha }_{max} }}$$

where α_*min*_ and α_*max*_ denotes the minimum and maximum thermal diffusivity of the sample. DI varies between 0 and 1, a value close to 0 means uniform filler dispersion.

**Figure 5:**
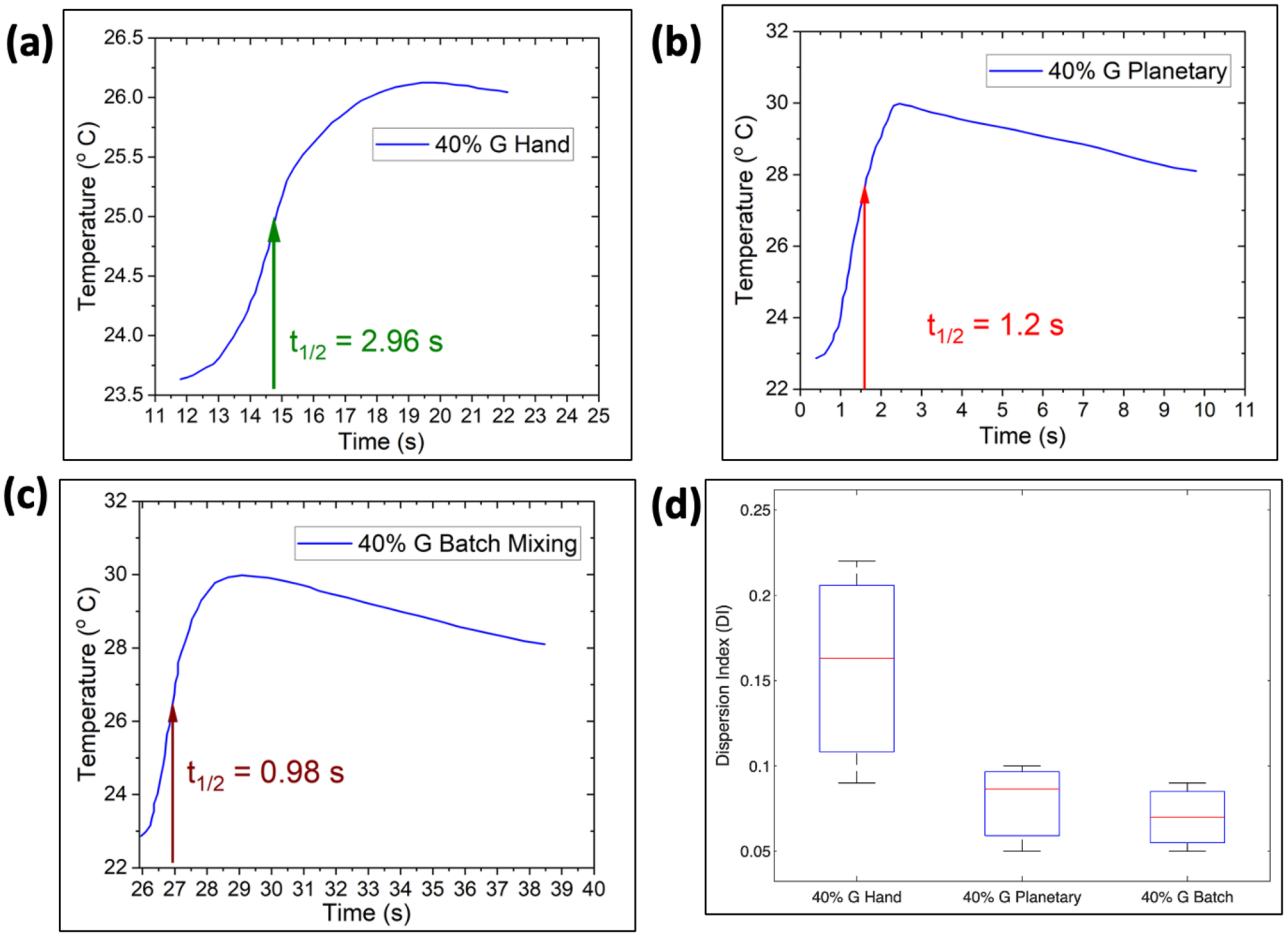
Thermal diffusivity of 40% G Ecoflex prepared by (**a**) hand mixing, (**b**) planetary mixing, (**c**) batch mixing, (**d**) dispersion index (DI) comparison of hand, planetary, and batch mixing.

Three different mixing methods (Hand, Planetary, and Batch mixing) were used to compare the dispersion of the samples and thus ensure the quality of homogeneity of these methods. By taking the average temperature over the whole sample area, the temperature profile is shown for 40% G hand, 40% G planetary and 40% G batch mixing samples respectively in Fig. 5a–c. The half rise time (t_1/2_) was calculated from the temperature profile. By measuring each pixel half time rise (t_1/2_), the thermal diffusivity in each pixel was calculated. To calculate the dispersion index for a specific area, the maximum and minimum thermal diffusivity is needed. Hence, we determined the dispersion by dividing the area into 4 squares. Figure 5d depicts the dispersion index with error bars for each of the samples. The mean dispersion index for hand, planetary, and batch mixing (whole sample) were 0.163, 0.0865, and 0.07, respectively. However, the hand mixing sample showed a wide range of dispersion at different position, and for planetary sample showed a dispersion index 0.04–0.10. Batch mixing sample depicts a narrower range in dispersion index meaning the dispersion of the fillers are nearly same (homogenous) in all over the composite. This ensures that batch mixing outperforms planetary in terms of filler homogeneity, while planetary outperforms hand-mixing.

### Curing and thermal characteristics of polymer composites

**Figure 6:**
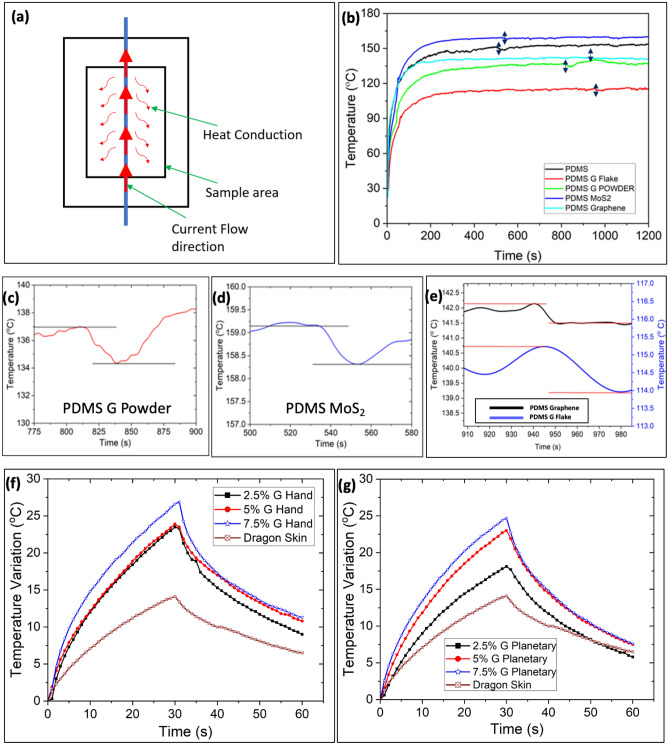
(**a**) Schematic of micro-scale curing analysis using nichrome wire in a 3D printed chamber. (**b**) Curing analysis of different fillers with PDMS polymer (vertical double arrow line depicts curing. Rapid temperature change during curing for (**c**) PDMS G powder, (**d**) PDMS MoS_2_, (**e**) PDMS graphene, and PDMS G flake. Thermal characteristics curve of dragon skin, 2.5%, 5%, 7.5% G Samples (each sample was heated by a SpotIR heater for 30 s and then cooled down for another 30 s) prepared by (**f**) Hand mixing and (**g**) Planetary mixing.

Polymer composites show a quick temperature change (exothermic reaction) signifying a transition from a viscous fluid to a solid during curing. A customized 3D printed chamber (3 × 4 × 2 mm^3^) was used as a sample holder and then constant heat was provided via the heater (Fig. 6a). The same Fluke RSE600 IR thermal camera with micro-lens was used to measure the curing and thermal characteristics phenomena. As a result, a higher resolution (20 µm/pixel) for each individual pixel was achieved to study the curing phenomenon in polymer composites. The trend for maximum temperature reached by Polydimethylsiloxane (PDMS)-MoS_2_, then PDMS without filler, and PDMS with larger filler respectively. So, curing can be determined when the constant maximum temperature deviates rapidly. While PDMS-Graphene and PDMS Graphite Flake (PDMS G Flake) cure in about 950 s, PDMS (without filler) takes 500 s to cure (Fig. 6b). MoS_2_ takes about 520 s to cure, compared to 800 s for PDMS Graphite Powder (PDMS G Powder). The rapid change in temperature or curing is shown in Fig. 6c–e for different composites. As a result, this method can show how fillers affect the curing of composites at the microscale, which is difficult to do with traditional techniques like differential scanning calorimetry.

Figure 6f,g shows the heating and cooling curve of polymer composites, and it provides the real-time average temperature over the whole sample area. Dragon skin polymer without fillers reaches a maximum temperature change of ~ 14 °C at 30 s from room temperature, whereas the temperature change increases (~ 24 to ~ 28 °C) when the graphite percentages increase from 2.5 to 7.5 at 30 s. This is due to the reason for filler addition in the polymer matrix (graphite has higher thermal conductivity than elastomer) and thus increases the total heat transfer into the composites. Agglomerated filler composites have different thermal conductivity than homogenous/well-dispersed composites. Well-dispersed graphite flake can transfer the heat to the polymer matrix and thus the average temperature decreases in planetary samples than in normal hand-mixing samples. A similar trend was also found from the thermal characteristics of 2.5%-7.5% G Planetary samples, but temperature change increases from ~ 17 to ~ 25 °C at 30 s. For a well-dispersed sample like planetary mixing, the temperature change at 30 s will be within a range for a specific weight percentage. So, obtaining the front surface temperature of the sample (either targeting a smaller surface area or a larger surface area) can indicate the quality of the sample. The thermal characteristics of two 40% G Ecoflex samples were also shown in Fig. S5. As the sample fabrication of the two samples was different, the temperature change (deviation of ~ 3.5 °C at 30 s) was different. As a result, thermal behavior employing infrared thermography and comparing it with a standard sample might be an excellent platform to examine good or bad quality composite samples in a manufacturing line.

## Conclusion

Thermal signatures from composites at the micro-scale can provide important information about micro/nanocomposite performance. In our experiment, using micro-scale thermal images and machine learning, we analyzed the dispersion, thermal diffusivity, curing, and dispersion index of graphite/graphene-based composites in the range from 2.5% to 40% weight ratio for different mixing techniques. For dispersion, batch mixing showed better homogeneity than planetary and hand mixing. The dispersion index for batch, planetary, and hand mixing for 40% graphite samples were 0.07, 0.0865, and 0.163 respectively. Tramp material/void/flaw detection in polymer composite was investigated with this machine learning-based model. Curing phenomenon of polymer with different fillers was also analyzed, and it has been shown that the curing time differs due to the filler type and size within the polymer matrix. Thus, infrared thermography integrating with our machine learning model (SDFN) can be a great non-destructive tool for aerospace and industrial applications where composite quality can be measured automatically both qualitatively and quantitatively in a real-time fashion. In future, we will extend this work to investigate in-depth defects or voids in a polymer composite.

## Supplementary Information

Supplementary Information.

## Data Availability

The datasets used and/or analysed during the current study available from the corresponding author on reasonable request.
