# Supplementary material for: Automatic dispersion, defect, curing, and thermal characteristics determination of polymer composites using micro-scale infrared thermography and machine learning algorithm
Source: Sci Rep. 2023 Feb 16;13:2787. doi: 10.1038/s41598-023-29270-z (PMC9935896; doi:10.1038/s41598-023-29270-z)
Supplement: Supplementary file 1 — Supplementary Information. [file 41598_2023_29270_MOESM1_ESM.docx]

# **Supplementary Information**

# **Automatic Surface Dispersion, Defect, Curing and Thermal Characteristics Determination of Polymer Composites using Infrared Thermography and Machine Learning Algorithm**

Md Ashiqur Rahman^1^, Mirza Masfiqur Rahman^2^, Ali Ashraf^1,*^

^1^Department of Mechanical Engineering, University of Texas Rio Grande Valley, Edinburg, TX-78539, USA.

^2^Department of Computer Sciences, Purdue University, West Lafayette, IN-47907, USA.

*Corresponding Author: Ali Ashraf (Email: ali.ashraf@utrgv.edu)


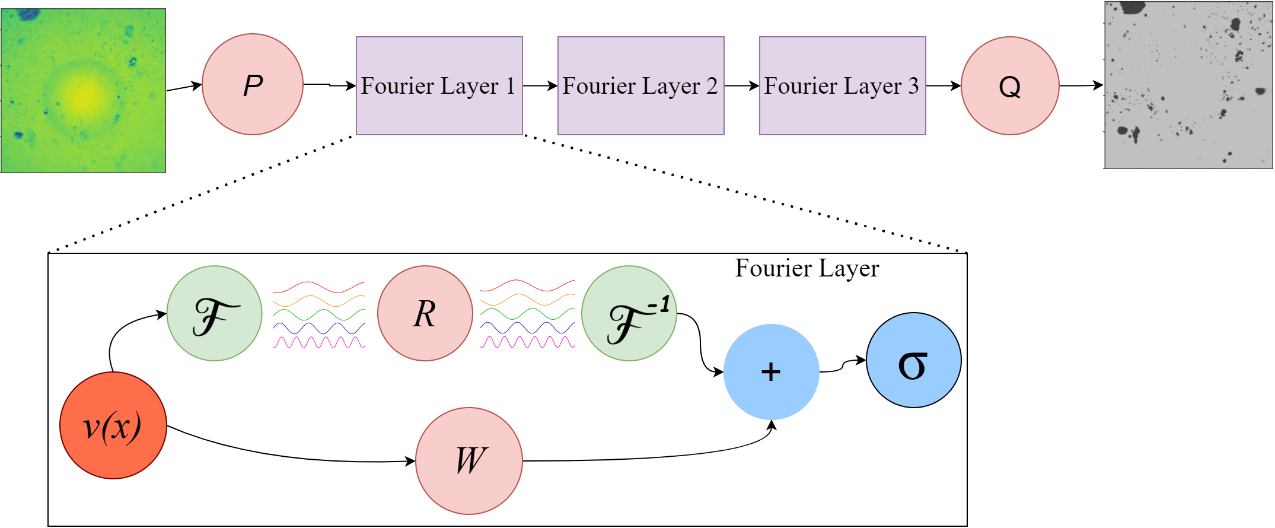


**Figure S1:** Schematic of SDFN architecture.


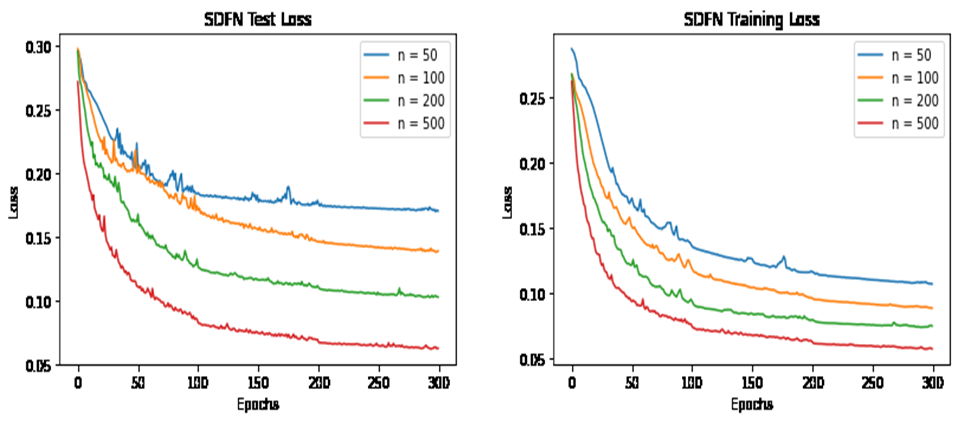


**Figure S2:** Test loss and training loss of SDFN model with varying number of runs.

**(a)**


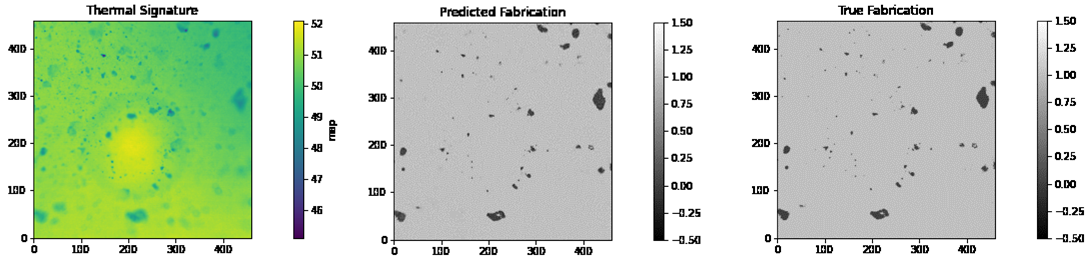


**(b)**


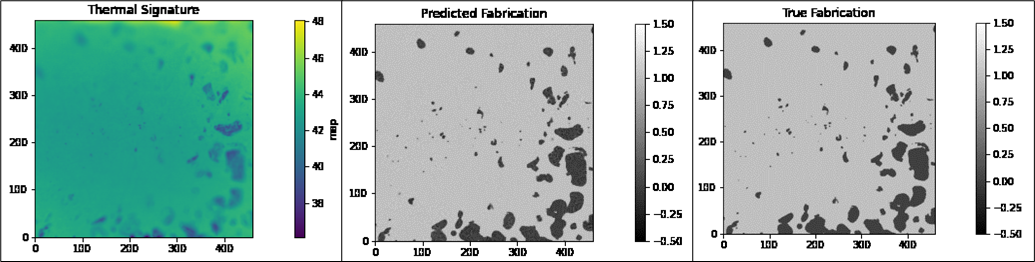


**(c)**


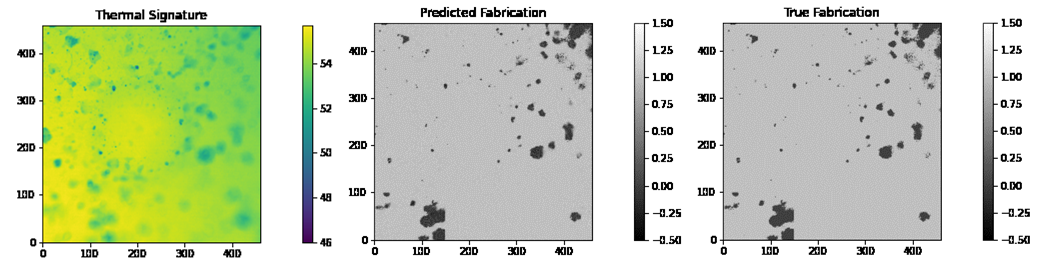


**(d)**


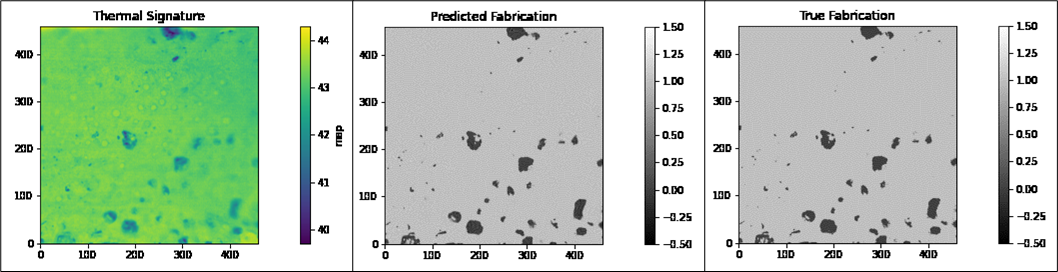


**Figure S3:** Thermal signature, predicted and true image of (a) 5% G Planetary, (b) 5% G Hand, (c) 7.5% G Hand, (d) 7.5% G Planetary.


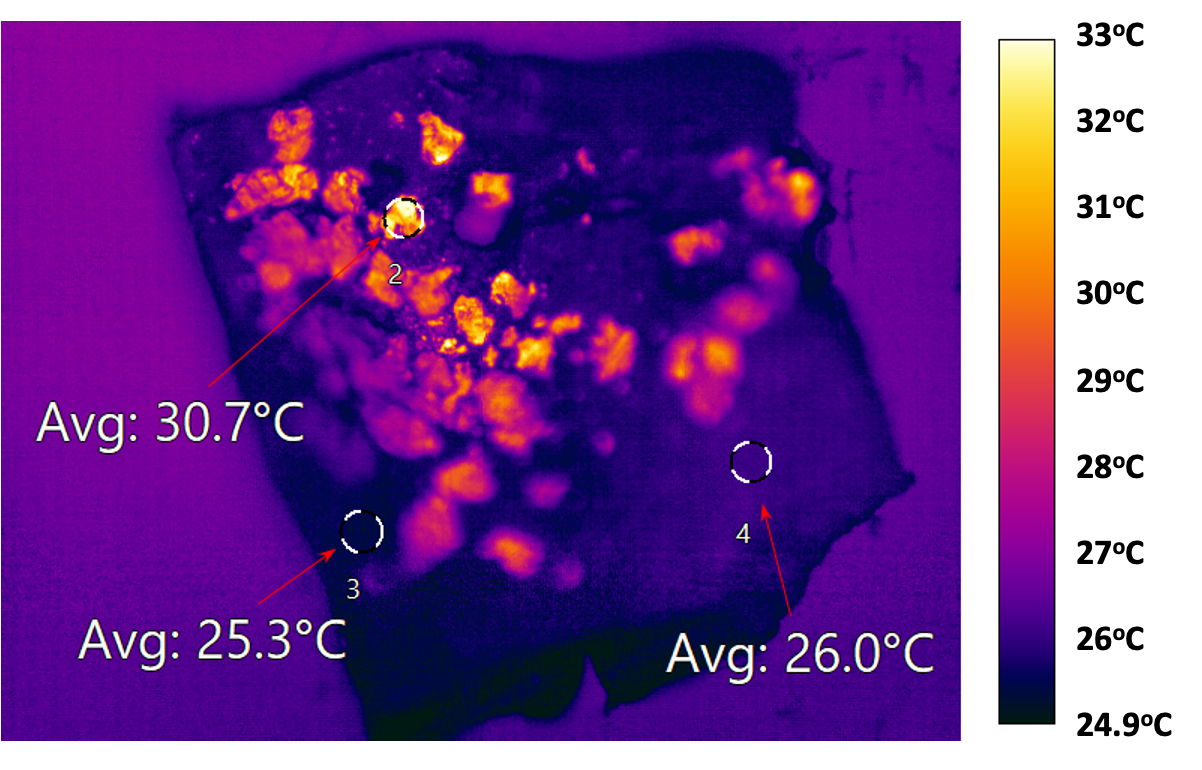
**Figure S4:** Filler identification (tramp material analysis) of polymer composites using MoS_2_, graphite, and ecoflex elastomer. The average temperature of each region was distinct due to emissivity.


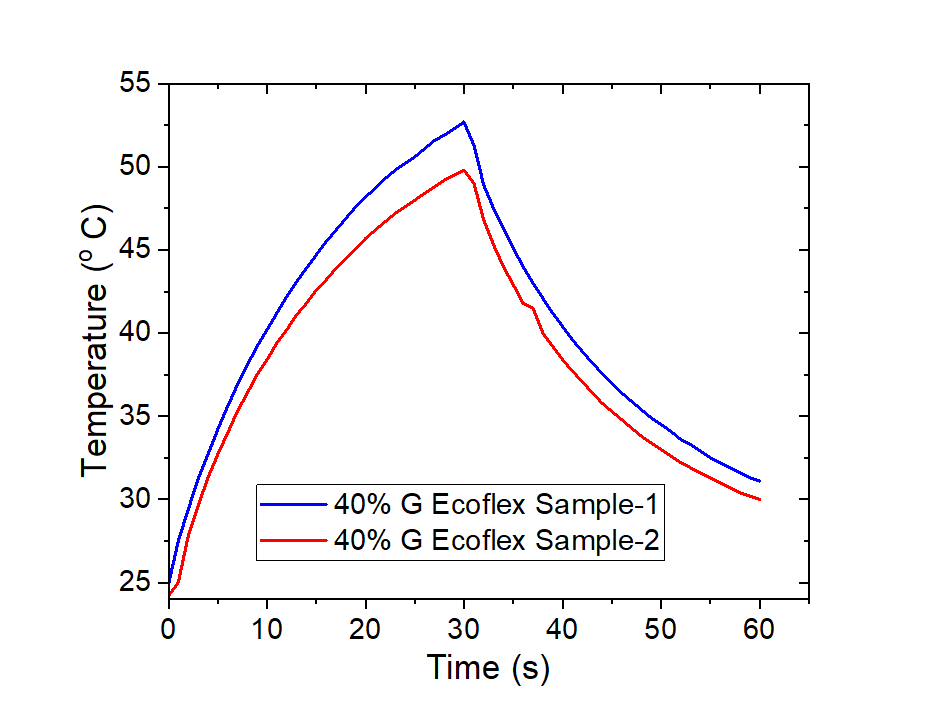
**Figure S5:** Thermal characteristics curve of 40% G Batch mixing samples.
